# Validation of single and combined screening tools for the detection of poststroke mood disorders in acute stroke patients in Singapore

**DOI:** 10.1186/s13030-025-00341-z

**Published:** 2025-10-08

**Authors:** Matthew H. S. Ng, Lai Gwen Chan

**Affiliations:** 1 Rehabilitation Research Institute of Singapore, Precision Rehabilitation, 11 Mandalay Road, #14-03 Clinical Science Building, Singapore, 308232 Singapore; 2 Department of Psychiatry, Tan Tock Seng Hospital, 11 Jalan Tan Tock Seng, Singapore, 308433 Singapore

**Keywords:** Stroke, Rehabilitation, Screening, Validation, Singapore, Poststroke depression

## Abstract

**Objective:**

Screening tools such as the Patient Healthcare Questionnaire (PHQ) and Hospital Anxiety and Depression Scale (HADS) are useful for detecting poststroke depression (PSD). However, validation of these tools has yet to be conducted in the Singapore stroke population.

**Method:**

A total of 138 adults were administered the HADS and both two- and nine-item versions of the PHQ. Receiver operating characteristic (ROC) analyses were utilized to validate these scales against the gold standard diagnosis of PSD through the Structured Clinical Interview for DSM-IV (SCID-IV).

**Results:**

All the tools examined in this study had good convergent validity (*r*s = 0.55–0.89) and discriminative power (Area Under Curve: 0.849–0.887). The optimal cut-off scores were ≥ 7 for the HADS depression and anxiety subscales, ≥ 10 for the total score, ≥ 2 for the PHQ-2 and ≥ 8 for the PHQ-9. Additional analyses suggest that the use of both the PHQ and the HADS in specific combinations further improved diagnostic performance.

**Conclusion:**

Both the PHQ and the HADS are acceptable tools for screening for poststroke depression in Singaporean inpatient rehabilitation care settings. Furthermore, our findings lead us to recommend using PHQ-9 with HADS-A for superior screening performance at sensitivity = 83.33%, specificity = 88.33%, positive predictive value = 51.72%, and negative predictive value = 97.25%.

## Introduction

 Poststroke depression (PSD) is a common neuropsychiatric sequela after stroke and is often associated with greater impairment, such as large reductions in activities of daily living [1], increased mortality [2] and poorer quality of life [3–4]. PSD also poses significant challenges to stroke recovery and rehabilitation [5–7] and has been shown to be associated with detrimental outcomes longitudinally [8–9], with widespread impact on families and caregivers [10–12]. Therefore, early detection and treatment of PSD are critical. Many researchers speculate that PSD is often underdiagnosed [14–15] in part because of difficulties such as cognitive impairment or communication deficits due to the stroke [16]. Furthermore, stroke often has many co-occurring somatic symptoms that overlap with depression, such as poor concentration, loss of appetite and fatigue, which often confound routine screenings to detect PSD [17].

 Structured clinical interviews have been established as the “gold standard” to assess and diagnose PSD. However, owing to practical challenges with administering structured clinical interviews in routine stroke care, many protocols and guidelines [11, 16, 18] have advocated for the utilization of screening tools to assess a larger number of patients efficiently, followed by a clinician’s diagnostic assessment of those who screened positive. Nevertheless, there is a lack of consensus on the most ideal screening tool to detect PSD across different healthcare and cultural settings [19–20]. This has caused difficulty in the comparative synthesis of results across different studies and has led to challenges in implementation in clinical practice. Various depression screening tools, including the Hospital Anxiety and Depression Scale (HADS) [21] and the Patient Health Questionnaire (PHQ) [22], which are of primary interest in this study, have been frequently utilized in the assessment of PSD [13, 23–27]. The HADS has been recommended because of the absence of items related to somatic symptoms that may lead to high rates of misclassification due to overlap with stroke symptoms, especially in the acute phase [28]. The PHQ, on the other hand, is known for its brevity, simplicity and free access to its use.

 There is a scarcity of studies in the extant literature validating the use of the PHQ and the HADS in Singapore. For instance, Sung and colleagues found that the sensitivity and specificity of the PHQ in detecting depression in a multiethnic sample of Asian primary care patients was 91.7% and 72.2%, respectively at a score of ≥ 6 [29]. Similarly, the HADS has currently been validated for use in Singapore cancer patients with a cut-off score of 7 on HADS-D and a cut-off score of 5 on HADS-A [30]. Despite the lack of validation, the HADS has been used in Singapore populations with rheumatoid arthritis [31], psoriasis [32] and HIV infection [33] using general recommended cut-off scores of 8 on either subscale. However, both PHQ and HADS have yet to be validated for stroke patients in Singapore and the lack of agreement on a suitable threshold score necessitates the conduct of this study. Moreover, in the authors’ clinical experience, PSD in the inpatient rehabilitation setting is commonly comorbid with neurological and anxiety symptoms, which renders the selection of a single optimal tool challenging. Hence, the present study seeks to evaluate the criterion validity of the HADS and both the two-item and nine-item versions of the PHQ (PHQ-2 and PHQ-9) against the gold standard Structured Clinical Interview for the DSM-IV (SCID) as screening instruments for the detection of PSD in Singaporean patients in the inpatient rehabilitation setting, as well as their convergent validity with each other. Additionally, we hypothesize that combining the HADS and PHQ would result in superior screening performance compared with that of either tool administered in silo.

## Methods

### Participants and procedures

 Data for this study were extracted from a standing database of stroke patients at a large tertiary hospital in Singapore and analysed with approval from the Domain-Specific Review Board. The extracted data were a subset of a larger database and included patients who underwent the validation phase of a pilot clinical project to implement poststroke depression screening into routine stroke rehabilitation care from 3 February 2017 to 6 September 2017. The participants of this project were screened for PSD during the inpatient rehabilitation phase of acute stroke. The inclusion criteria for screening were as follows: participants were not delirious by clinical judgement, were not aphasic and were able to complete the tools independently or with assistance from trained allied health professionals. The participants were screened with the English versions of the PHQ-9, the HADS and the SCID. A priori power analysis revealed that to detect an effect of at least 0.8 or more, at the Type I error rate of 0.05, at least 13 cases were required in the positive group [34]. One hundred and thirty-eight consecutive stroke patients (*N* = 138), which included 18 positive cases as diagnosed by the SCID, underwent this procedure during the validation period.

## Measures

### Patient health questionnaire

 The PHQ-9 is a brief, self-administered nine-item depression screening tool based on the nine items in the DSM-IV diagnostic criteria for major depression. Each item can be scored from 0 to 3, with a total score ranging from 0 to 27, with higher scores indicating greater symptom severity and a threshold score of 10 or more had a sensitivity and specificity of 88% for major depression in primary care patients [22].

 The PHQ-2 is derived from the PHQ-9 and comprises two questions related to the two core criteria for major depression – pervasive low mood and anhedonia in the last two weeks. A cut-off score of 3 or more had a sensitivity of 83% and specificity of 92% for major depression [35]. Specifically, in a multicentre study of the stroke population [25], administering the PHQ-9 only to patients who scored ≥ 2 on the PHQ-2 improved the sensitivity of the PHQ-9 at a threshold of ≥ 10.

### Hospital anxiety and depression scale (HADS)

 The HADS, developed by Zigmond and Snaith [21], is a 14-item self-administered scale comprising 7 items in the anxiety subscale (HADS-A) and 7 items in the depression subscale (HADS-D). Each item has a score range of 0–3, and subscale scores are obtained by summing all the items within each subscale. Each subscale ranges from 0 to 21, with higher scores indicating greater severity of symptoms.

 The depression subscale scores (HADS-D) and total scores (HADS-T) (obtained by summing both anxiety and depression subscale scores) have been used for screening for PSD, with studies identifying the thresholds of the HADS-D and HADS-T to be between 4 and 8 [23, 26–28], and 14, respectively [27]. However, the utility of HADS-A in detecting PSD has not been examined. Our study specifically examined this because clinical experience indicates that anxious distress is a common comorbidity in depressed patients in the hospital setting.

### Structured clinical interview for the DSM-IV (SCID)

 For this study, the PSD screening tools were validated against the DSM-IV criteria for Mood Disorder Due to a General Medical Condition (Stroke) as the “gold standard” for diagnosing PSD. Diagnoses were determined by a psychiatrist administering the SCID to detect major depression, minor depression, and adjustment disorders in patients who completed the PHQ and HADS. Diagnostic outcomes were then categorized as “any PSD” for patients experiencing any form of mood disorder and “no PSD” for patients who were not diagnosed with any disorder.

### Demographic data and stroke characteristics

 The demographic variables extracted included age, sex, ethnicity, marital status, education level, history of psychiatric disorders and occupational status. Stroke laterality, nature, and incidence were also extracted.

### Analytic plan

 Statistical analysis was performed via the Statistical Package for Social Sciences (SPSS) for Windows version 25.0 [36]. The internal consistency of each tool was measured with Cronbach’s alpha and McDonald’s omega coefficient, and convergent validity of the tools was examined via Pearson’s correlation analysis. Next, for criterion validity against the SCID, receiver operating characteristic (ROC) curves with area under the curve (AUC) statistics were generated for HADS-A, HADS-D, HADS-T, PHQ-9, and PHQ-2. Finally, tables comparing the sensitivity, specificity, positive predictive value (PPV), and negative predictive value (NPV) were generated for potential threshold scores.

## Results

### Participant demographics

 Data from 138 participants, 69% of whom were male and between 40 and 86 years of age (mean 62.76, SD 10.68), were analysed, and their characteristics are presented in Table 1. Most of the participants were ethnically Chinese (86.2%), employed (51.5%) and had at least primary school education (55.1%). As shown in Table 1 18 out of 138 (13.0%) patients were diagnosed with a poststroke mood disorder through the SCID, specifically, 5 patients were diagnosed with post-stroke depression and 13 were diagnosed with adjustment disorder. Among these 18 patients, 3 had a history of depressive disorder and 1 had a non-mood disorder psychiatric history and were included in the primary analysis. The PHQ-9, HADS-A and HADS-D screening tools showed acceptable to good internal consistency, with a Cronbach’s alpha (α) between 0.766 and 0.83; (ω = 0.82–0.91), meaning that the items within each test measure the same construct of mood disorders consistently [37]. However, only the PHQ-2 demonstrated a questionable level of internal consistency (*α* = ω = 0.66).

**Table 1:** Summary of participant characteristics

	Demographic Characteristic	*n*	Mean (SD) or %	Range		
	Age	138	62.76 (10.68)	40	-	86
**Sex**						
	Male	95	68.84%			
	Female	43	31.16%			
**Race/Ethnicity**						
	Chinese	119	86.23%			
	Non-Chinese	19	13.77%			
**Marital Status**						
	Married	86	62.32%			
	Single	52	37.68%			
**Education**						
	< 10 years of formal education	62	44.93%			
	> 10 years of formal education	76	55.07%			
**Occupation**						
	Unemployed	67	48.55%			
	Employed	71	51.45%			
**Psychiatric History**						
	History of any psychiatric disorder	4	2.90%			
	No history of psychiatric disorders	134	97.10%			
**Stroke Laterality**						
	Left	55	39.86%			
	Right	69	50.00%			
	Bilateral	14	10.14%			
**Stroke Incidence**						
	First Stroke	57	41.30%			
	First stroke with silent strokes on neuroimaging	58	42.03%			
	Recurrent	23	16.67%			
**Stroke Nature**						
	Ischemic	110	79.71%			
	Haemorrhagic	28	20.29%			
**Variables of Interest**						
	SCID Positive	18	13.04%			
	PHQ-2	138	1.31 (1.70)	0	-	6
	PHQ-9	138	4.99 (4.94)	0	-	26
	HADS-D	137	3.16 (4.03)	0	-	17
	HADS-A	137	2.74 (4.00)	0	-	20

### Convergent validity

 Pearson’s correlation coefficient was used to examine the convergent validity of the screening tools. As shown in Table 2, all tools showed relatively moderate to high Pearson’s correlations (*rs* = 0.548–0.887, *ps* < 0.01), indicating that mood disorder severity scores across all four tools presented moderate to strong associations.

**Table 2:** Convergent validity of the screening tools utilized in this study

	Name	1	2	3	4
1	PHQ-2				
2	PHQ-9	0.772^***^	-		
3	HADS-D	0.666^***^	0.719^***^	-	
4	HADS-A	0.548^***^	0.664^***^	0.574^***^	-
5	HADS-T	0.680^***^	0.776^***^	0.887^***^	0.887***

Note. *p < .05; **p < .01; ***p < .001

### Performance of the hospital anxiety and depression scale

 The HADS-A showed good criterion validity according to the ROC analysis, with an AUC of 0.803 (95% CI 0.685–0.920, *p <* .001). The optimal cut-off for HADS-A was a score ≥ 7, reflecting optimal sensitivity, specificity, PPV and NPV. Furthermore, the HADS-A had good internal consistency (α = 0.83, ω = 0.89). The HADS-D subscale also showed good criterion validity based on the ROC curve analysis, with an AUC of 0.859 (95% CI 0.775–0.944, *p <* .001). The optimal cut-off for the HADS-D score was a score of ≥ 7, balancing sensitivity, specificity, PPV and NPV. Furthermore, the HADS-D also had good internal consistency (α = 0.79, ω = 0.91). Finally, strong validity was shown for the HADS-T, with an AUC of 0.849 (95% CI 0.749–0.950, *p <* .001) in the ROC analysis. From this analysis, a threshold score of ≥ 10 and above gives the optimal sensitivity, specificity, PPV and NPV. The HADS-T also demonstrated good internal consistency (α = 0.88, ω = 0.92).

### Performance of patient healthcare questionnaire

**Fig. 1:**
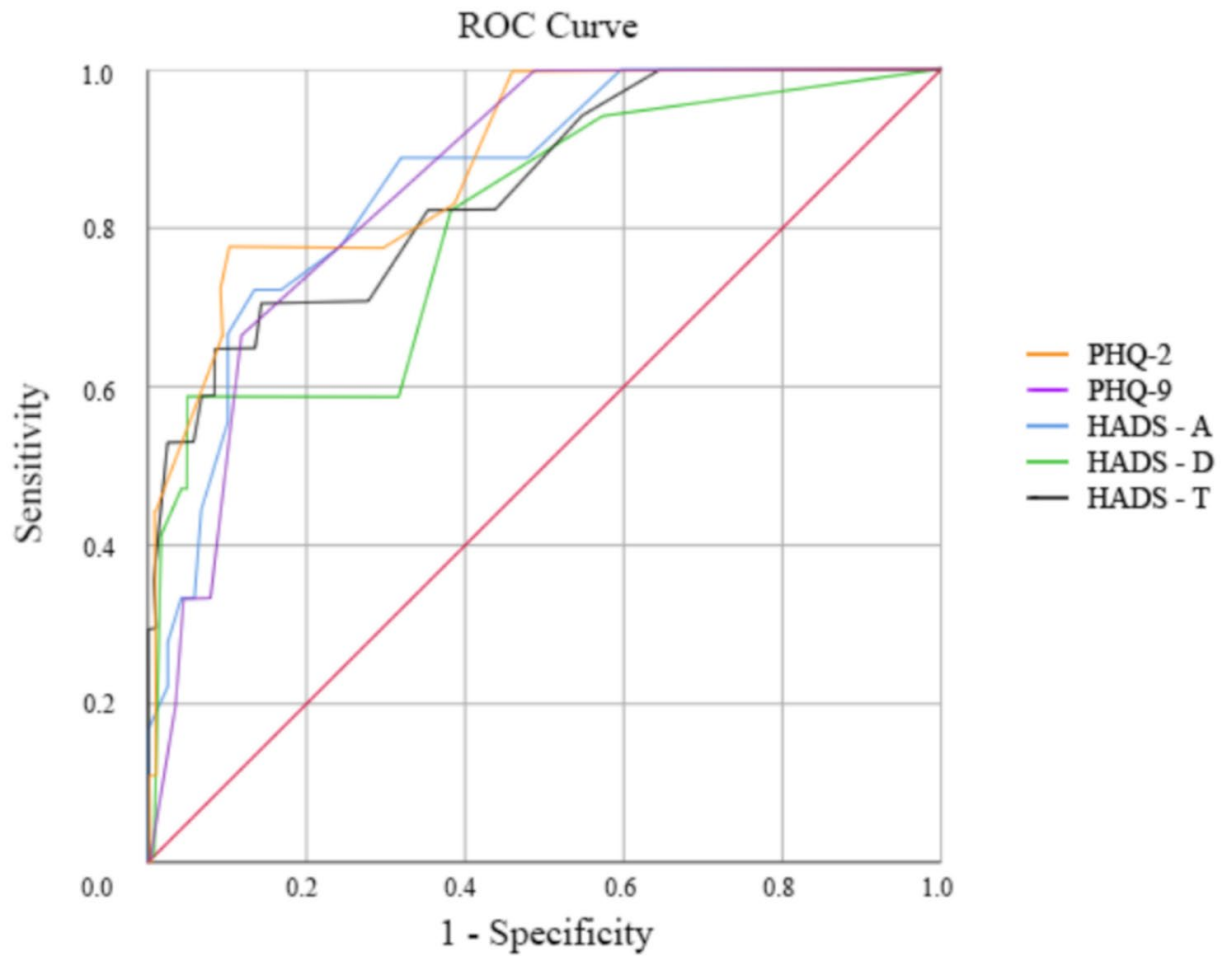
Receiver operating characteristic curves of the PHQ-2, PHQ-9, and HADS subscales and total scores in detecting poststroke depression

 The results of the ROC analysis revealed that the PHQ-9 had an AUC of 0.887 (95% CI 0.808–0.966, *p* < 0.001), with an optimal cut-off score of ≥ 8 indicating the best sensitivity, specificity, PPV and NPV. The PHQ-9 also had acceptable internal consistency (α = 0.766, ω = 0.82). The PHQ-2 displayed good criterion validity, with the ROC analysis reporting an AUC of 0.857, 95% CI 0.782 to 0.932, *p* < 0.001. The recommended cut-off of 2 or more was shown to have optimal sensitivity, specificity and NPV but at the expense of PPV. This finding is also in line with other studies in the stroke population [24]. Refer to Fig. 1; Table 3 for a summary of all ROC curve analyses and optimal cut-offs, respectively.

**Table 3:** Diagnostic utility of the PHQ2, PHQ9, HADS-A, HADS-D and HADS-T across a range of threshold scores

Screening Tool	Threshold score	Sensitivity	Specificity	PPV	NPV
HADS-A	≥ 5	55.56%	85.00%	35.71%	92.73%
	≥ 6	55.56%	89.17%	43.48%	93.04%
	**≥ 7**	**55.56%**	**95.00%**	**62.50%**	**93.44%**
	≥ 8	50.00%	95.00%	60.00%	92.68%
	≥ 9	44.44%	95.00%	57.14%	91.94%
HADS-D	≥ 5	72.22%	83.33%	39.39%	95.24%
	≥ 6	72.22%	86.67%	44.83%	95.41%
	**≥ 7**	**66.67%**	**90.00%**	**50.00%**	**94.74%**
	≥ 8	55.56%	90.00%	45.45%	93.10%
	≥ 9	44.44%	93.33%	50.00%	91.80%
HADS-T	≥ 8	66.67%	83.33%	37.50%	94.34%
	≥ 9	66.67%	85.00%	40.00%	94.44%
	**≥ 10**	**66.67%**	**85.83%**	**41.38%**	**94.50%**
	≥ 11	61.11%	86.67%	40.74%	93.69%
	≥ 12	61.11%	88.33%	44.00%	93.81%
PHQ-9	≥ 6	77.78%	70.83%	28.57%	95.51%
	≥ 7	77.78%	81.67%	38.89%	96.08%
	**≥ 8**	**77.78%**	**90.00%**	**53.85%**	**96.43%**
	≥ 9	72.22%	90.83%	54.17%	95.61%
	≥ 10	66.67%	90.83%	52.17%	94.78%
PHQ-2	≥ 1	100.00%	51.67%	23.68%	100.00%
	**≥ 2**	**77.78%**	**74.17%**	**31.11%**	**95.70%**
	≥ 3	66.67%	88.33%	46.15%	94.64%

Note: Bolded rows indicate the optimal cut-offs for each of the tools

### Diagnostic value of tools in combination

 In addition to determining the diagnostic utility of the individual screening tools, further analysis was performed to examine the diagnostic utility of these tools in combination to explore whether administering the tools in combination might improve their utility in detecting PSD, commensurate with the authors’ clinical experience. Sensitivity, specificity, NPV and PPV analyses were performed with a combination of screening tools, as shown in Table 4. The results of these analyses reveal possible combinations of screening tools that could be utilized depending on whether the priority is sensitivity/specificity or positive and negative predictive values. For the best improvement in sensitivity, meeting the cut-off score on either the PHQ (both 2 and 9-item) or the HADS-A led to a sensitivity of > 83.0%. None of the combinations had an improved specificity beyond the HADS-A alone, nor a clear improvement in NPV beyond the PHQ-9 alone. However, the best improvement in PPV was provided by a positive screen on both PHQ-9 and HADS-T at 68.8%. Taken together, depending on the goal of screening (maximizing sensitivity/specificity or PPV/NPV), using both the PHQ and HADS in screening appears to improve the detection of PSD, as depressive and comorbid anxiety symptoms are captured.

**Table 4:** Diagnostic utility of individual scales and a combination of scales for detecting PSD

Screening Tool	Sensitivity	Specificity	PPV	NPV
HADS-A ≥ 7	55.56%	95.00%	62.50%	93.44%
HADS-D ≥ 7	66.67%	90.00%	50.00%	94.74%
HADS-T ≥ 10	66.67%	85.83%	41.38%	94.50%
PHQ-9 ≥ 8	77.78%	90.00%	53.85%	96.43%
PHQ-2 ≥ 2	77.78%	74.17%	31.11%	95.70%
**PHQ-2 ≥ 2 and HADS-A ≥ 7**	**50.00%**	**95.83%**	**64.29%**	**92.74%**
PHQ-2 ≥ 2 and HADS-D ≥ 7	61.11%	94.17%	61.11%	94.17%
PHQ-2 ≥ 2 and HADS-T ≥ 10	55.56%	90.00%	45.45%	93.10%
PHQ-9 ≥ 8 and HADS-A ≥ 7	61.11%	91.67%	52.38%	94.02%
PHQ-9 ≥ 8 and HADS-D ≥ 7	50.00%	97.50%	75.00%	92.86%
**PHQ-9 ≥ 8 and HADS-T ≥ 10**	**61.11%**	**95.83%**	**68.75%**	**94.26%**
**PHQ-2 ≥ 2 or HADS-A ≥ 7**	**88.89%**	**73.33%**	**33.33%**	**97.78%**
PHQ-2 ≥ 2 or HADS-D ≥ 7	83.33%	69.17%	28.85%	96.51%
PHQ-2 ≥ 2 or HADS-T ≥ 10	88.89%	70.00%	30.77%	97.67%
**PHQ-9 ≥ 8 or HADS-A ≥ 7**	**83.33%**	**88.33%**	**51.72%**	**97.25%**
PHQ-9 ≥ 8 or HADS-D ≥ 7	77.78%	84.17%	42.42%	96.19%
PHQ-9 ≥ 8 or HADS-T ≥ 10	83.33%	84.17%	44.12%	97.12%

Note: Bolded rows indicate the ideal screening tools utilized in combination

## Discussion

 Both the PHQ and the HADS have been validated internationally to screen for PSD [23–28, 38]. However, this study is the first to validate these depression screening questionnaires in a sample of consecutive Singaporean stroke patients in an inpatient rehabilitation setting. The PHQ-2, PHQ-9 and HADS were demonstrated to be acceptable tools for screening for PSD, featuring good convergent validity (*r*s = 0.55–0.89) and discriminative power (AUC: 0.849–0.887).

 Analyses revealed that the optimal cut-off scores for the HADS subscales were ≥ 7 for the HADS anxiety subscale and depression subscale and ≥ 10 for the HADS-T. The cut-offs for the two-item and nine-item versions of the PHQ were ≥ 2 and ≥ 8, respectively. These optimal threshold scores are in line with similar findings in previous validation studies to detect PSD with the HADS-D (4–7) [27, 28, 39], HADS-T (≥ 11) [27, 39], PHQ-9 (6–10) [24, 27] and PHQ-2 (≥ 2) [25, 27]. These findings have important clinical implications. Specifically, the validation of these instruments now allows for the screening of PSD in the Singaporean stroke population in the rehabilitation setting to be administered quickly and accurately without the need for a trained medical health professional.

 Furthermore, our analysis of the diagnostic performance of the tools used in combination revealed that when used together, these tools were more effective at detecting PSD than when used individually. Thus, while being mindful that there is a possibility of inflated reliability estimates due to significant convergent validity of the tools, one key recommendation made from this study is to administer both the PHQ and HADS in combination and identify patients who screen positive on these screening tools for further clinical diagnostic evaluation. This approach has been successfully implemented in routine stroke care at the authors’ institution. Clinical experience has proven this to be beneficial as the PHQ is limited by the omission of anxiety symptoms and potential overlap with somatic symptoms directly related to the stroke, and the HADS is limited by its focus on only psychological symptoms and its short symptom duration requirement that falls short of full diagnostic criteria for mood disorders. The symptom profile identified by the questionnaire responses also guides a more focused clinical interview to elicit important information that informs the eventual treatment plan. Patients who do not screen positive on at least one of either the PHQ-9 or HADS-A means that PSD can be effectively ruled out for them since the NPV is 97.3%. However, as the PPV for this screening combination is low at 51.7%, a more detailed diagnostic interview should be performed as there may be other poststroke sequelae that mimic PSD such as apathy. This is consistent with our clinical practice and experience.

 Our study has acceptable internal validity for a few reasons. First, the sample used in this study included a representative range of stroke types, such as ischemic, haemorrhagic, and recurrent strokes, even patients who required more time for initial poststroke delirium or aphasia to improve. Aside from these broad inclusion criteria, the main exclusion criterion is the inability to complete the tools despite assistance even on repeat attempts during the hospitalization stay. This reduces selection bias of the study sample and helps to avoid the confounding of PSD by significant cognitive impairment. The second reason is the selection of clinician-administered SCID as the gold standard comparison for criterion validity which reduces observer and measurement bias. Hence, this study also has good external validity as its results are generalizable across to the hospitalized stroke population in Singapore.

 Study limitations include the lack of a measure of interrater reliability and blinding of the screening and diagnostic outcomes to the raters, potentially contributing to the observed agreement. As the process of validation was run concurrently with routine clinical care, it was unfeasible for multiple healthcare staff to screen the same patient and be blinded when conducting diagnostic interviews. Furthermore, the study had a small sample size which may explain the low prevalence rate of PSD in the rehabilitation setting (13%) compared with previous studies [40] (25.4%). On the other hand, the lack of formal cognitive or neuropsychological assessments potentially leads to over-detection of PSD as mild poststroke cognitive impairments may influence self-reported mood symptoms. This may result in a lower precision of the accuracy statistics.

 As the current study was focused on examining the utility of screening tools in detecting the presence of mood disorders post-stroke, we did not assess the validity in the detection of specific mood disorders (i.e., minor depression, major depression or adjustment disorders). This would require future research with a larger sample size. In addition to screening for PSD, future work should be done to determine the accuracy of screening tools to detect other important neuropsychiatric sequelae after stroke, such as anxiety and cognitive impairment.

### Implications for behavioral health

 Nevertheless, given the accuracy and brevity of both the HADS and the PHQ, both scales are acceptable for screening PSD in the Singaporean rehabilitation care setting. Additionally, we recommend screening patients via both PHQ and HADS in combination to further improve the diagnostic performance of these tools. In our clinical experience, this is feasible and practical for both patients and healthcare teams. Specifically, this combination has been embedded into the standardized process of the ongoing poststroke depression screening program in the authors’ institution, and the program has shown a positive impact on improving the long-term clinical outcomes of stroke patients regardless of depression status [41]. 

## Data Availability

The datasets used and/or analysed during the current study are available from the corresponding author upon reasonable request.

## References

[1] Schmid AA, Van Puymbroeck M, Knies K, et al. Fear of falling among people who have sustained a stroke: a 6-month longitudinal pilot study. Am J Occup Ther. 2011;65(2):125–32. 10.5014/ajot.2011.000737 21476359 10.5014/ajot.2011.000737

[2] House A, Knapp P, Bamford J, Vail A. Mortality at 12 and 24 months after stroke May be associated with depressive symptoms at 1 month. Stroke. 2001;32(3):696–701. 10.1161/01.str.32.3.696 11239189 10.1161/01.str.32.3.696

[3] Hilari K, Lamping DL, Smith SC, et al. Psychometric properties of the stroke and aphasia quality of life scale (SAQOL-39) in a generic stroke population. Clin Rehabil. 2009;23(6):544–57. 10.1177/0269215508101729 19447841 10.1177/0269215508101729

[4] Kim ES, Kim JW, Kang HJ, et al. Longitudinal impact of depression on quality of life in stroke patients. Psychiatry Invest. 2017;15(2):141–6. 10.30773/pi.2017.10.11 10.30773/pi.2017.10.11 PMC5900407 29475223

[5] Paolucci S, Antonucci G, Pratesi L, Traballesi M, Grasso MG, Lubich S. Poststroke depression and its role in rehabilitation of inpatients. Arch Phys Med Rehabil. 1999;80(9):985–90. 10.1016/S0003-9993(99)90048-5 10488996 10.1016/s0003-9993(99)90048-5

[6] Schubert DSP, Taylor C, Lee S, Mentari A, et al. Detection of depression in the stroke patient. Psychosomatics. 1992;33(3):290–4. 10.1016/S0033-3182(92)71967-7 1410202 10.1016/S0033-3182(92)71967-7

[7] van de Weg FB, Kuik DJ, Lankhorst GJ. Poststroke depression and functional outcome: a cohort study investigating the influence of depression on functional recovery from stroke. Clin Rehabil. 1999;13(3):268–72. 10.1191/026921599672495022 10392654 10.1191/026921599672495022

[8] Kapoor A, Lanctot KL, Bayley M, et al. Screening for Post-Stroke depression and cognitive impairment at baseline predicts Long-Term Patient-Centered outcomes after stroke. J Geriatr Psychiatr Neurol. 2019;32(1):40–8. 10.1177/0891988718819859 10.1177/0891988718819859 30793663

[9] Kim ES, Kim JW, Kang HJ, et al. Longitudinal impact of depression on quality of life in stroke patients. Psychiatry Investig. 2017;15(2):141–6. 10.30773/pi.2017.10.11 29475223 10.30773/pi.2017.10.11 PMC5900407

[10] Dou DM, Huang LL, Dou J, Wang XX, Wang PX. Poststroke depression as a predictor of caregivers burden of acute ischemic stroke patients in China. Psychol Health Med. 2018;23(5):541–7. 10.1080/13548506.2017.1371778 28851230 10.1080/13548506.2017.1371778

[11] Gaete JM, Bogousslavsky J. Poststroke depression. Expert Rev Neurother. 2008;8(1):75–92. 10.1586/14737175.8.1.75 18088202 10.1586/14737175.8.1.75

[12] Gainotti G, Azzoni A, Marra C. Frequency, phenomenology and anatomical–clinical correlates of major poststroke depression. Br J Psychiatry. 1999;175(2):163–7. 10.1192/bjp.175.2.163 10627800 10.1192/bjp.175.2.163

[13] Williams CL, Rittman MR, Boylstein C, et al. Qualitative and quantitative measurement of depression in veterans recovering from stroke. J Rehabilitation Res Dev. 2005;42(3):277–90. 10.1682/jrrd.2004.02.0017 10.1682/jrrd.2004.02.0017 16187241

[14] Schulberg HC, Saul M, McClelland M, et al. Assessing depression in primary medical and psychiatric practices. Arch Gen Psychiatry. 1985;42(12):1164–70. 10.1001/archpsyc.1985.01790350038008 4074109 10.1001/archpsyc.1985.01790350038008

[15] Rogers SC. Poststroke depression screening: an executive summary. J Neurosci Nurs. 2017;49(2):66–8. 10.1097/JNN.0000000000000270 28277448 10.1097/JNN.0000000000000270

[16] Turner-Stokes L, Hassan N. Depression after stroke: a review of the evidence base to inform the development of an integrated care pathway. Part 1: Diagnosis, frequency and impact. Clin Rehabil. 2002;16(3):231–47. 10.1191/0269215502cr487oa 12017511 10.1191/0269215502cr487oa

[17] Staub F, Bogousslavsky J. Poststroke depression or fatigue. Eur Neurol. 2001;45(1):3–5. 10.1159/000052081 11205620 10.1159/000052081

[18] Bowen A, James M, Young G. National clinical guideline for stroke: Fifth Edition 2016.

[19] Gall A. Poststroke depression. Br J Therapy Rehabilitation. 2001;8(7):6.

[20] Meader N, Moe-Byrne T, Llewellyn A, ET AL. Screening for poststroke major depression: a meta-analysis of diagnostic validity studies. J Neurol Neurosurg Psychiatry. 2014;85(2):198–206. 10.1136/jnnp-2012-304194 23385849 10.1136/jnnp-2012-304194

[21] Zigmond AS, Snaith RP. The hospital anxiety and depression scale. Acta Psychiatrica Scandinavica. 1983;67(6):361–70. 10.1111/j.1600-0447.1983.tb09716.x 6880820 10.1111/j.1600-0447.1983.tb09716.x

[22] Kroenke K, Spitzer RL, Williams JBW. The PHQ-9: validity of a brief depression severity measure. J Gen Intern Med. 2001;16(9):606–13. 10.1046/j.1525-1497.2001.016009606.x 11556941 10.1046/j.1525-1497.2001.016009606.x PMC1495268

[23] Aben I, Verhey F, Lousberg R, et al. Validity of the Beck depression Inventory, hospital anxiety and depression scale, SCL-90, and Hamilton depression rating scale as screening instruments for depression in stroke patients. Psychosomatics. 2002;43(5):386–93. 10.1176/appi.psy.43.5.386 12297607 10.1176/appi.psy.43.5.386

[24] Dajpratham P, Pukrittayakamee P, Atsariyasing W, et al. The validity and reliability of the PHQ-9 in screening for poststroke depression. BMC Psychiatry. 2020;20(1):291. 10.1186/s12888-020-02699-6 32517743 10.1186/s12888-020-02699-6 PMC7285729

[25] de Man-van Ginkel JM, Hafsteinsdóttir T, Lindeman E, et al. An efficient way to detect poststroke depression by subsequent administration of a 9-Item and a 2-Item patient health questionnaire. Stroke. 2012;43(3):854–6. 10.1161/STROKEAHA.111.640276 22156689 10.1161/STROKEAHA.111.640276

[26] O’Rourke S, MacHale S, Signorini D, et al. Detecting psychiatric morbidity after stroke: comparison of the GHQ and the HAD scale. Stroke. 1998;29(5):980–5. 10.1161/01.STR.29.5.980 9596246 10.1161/01.str.29.5.980

[27] Turner A, Hambridge J, White J, et al. Depression screening in stroke: a comparison of alternative measures with the structured diagnostic interview for the diagnostic and statistical manual of mental disorders, fourth edition (major depressive episode) as criterion standard. Stroke. 2012;43(4):1000–5. 10.1161/STROKEAHA.111.643296 22363064 10.1161/STROKEAHA.111.643296

[28] Kang HJ, Stewart R, Kim JM, et al. Comparative validity of depression assessment scales for screening poststroke depression. J Affect Disord. 2013;147(1–3):186–91. 10.1016/j.jad.2012.10.03 23167974 10.1016/j.jad.2012.10.035

[29] Sung SC, Low CCH, Fung DSS, et al. Screening for major and minor depression in a multiethnic sample of Asian primary care patients: A comparison of the nine-item patient health questionnaire (PHQ-9) and the 16-item quick inventory of depressive Symptomatology - Self-Report (QIDS-SR 16): depression screening in primary care. Asia-Pacific Psychiatry. 2013;5(4):249–58. 10.1111/appy.12101 24123813 10.1111/appy.12101

[30] Beck KR, Tan SM, Lum SS, et al. Validation of the emotion thermometers and hospital anxiety and depression scales in singapore: screening cancer patients for distress, anxiety and depression. Asia-Pac J Clin Oncol. 2016;12(2):e241–9. 24673756 10.1111/ajco.12180

[31] Ho RCM, Fu EHY, Chua ANC, et al. Clinical and psychosocial factors associated with depression and anxiety in Singaporean patients with rheumatoid arthritis. Int J Rheum Dis. 2011;14(1):37–47. 10.1111/j.1756-185X.2010.01591.x 21303480 10.1111/j.1756-185X.2010.01591.x

[32] Tee SI, Lim ZV, Theng CT, et al. A prospective cross-sectional study of anxiety and depression in patients with psoriasis in Singapore. J Eur Acad Dermatol Venereol. 2016;30(7):1159–64. 10.1111/jdv.1361 27027485 10.1111/jdv.13615

[33] Chan LG, Ho MJ, Kaur P, et al. Differences in clinical and psychiatric outcomes between prevalent HIV-1 molecular subtypes in a multiethnic Southeast Asian sample. Gen Hosp Psychiatry. 2016;38:4–8. 26380875 10.1016/j.genhosppsych.2015.07.008

[34] MedCalc Software Ltd. Sample size for Area under ROC curve. https://www.medcalc.org/calc/sample-size-area-under-roc-curve.php (Version 23.2.8; accessed July 20, 2025).

[35] Kroenke K, Spitzer RL, Williams JB. The patient health Questionnaire-2: validity of a two-item depression screener. Med Care. 2003;41(11):1284–92. 14583691 10.1097/01.MLR.0000093487.78664.3C

[36] IBM. SPSS statistics for Windows. Version 25.0. Armonk. NY: IBM; 2012.

[37] Tavakol M, Dennick R. Making sense of cronbach’s alpha. Intern J Med Educ. 2011;2:53–5. 10.5116/ijme.4dfb.8dfd 10.5116/ijme.4dfb.8dfd PMC4205511 28029643

[38] Johnson G, Burvill PW, Anderson CS, et al. Screening instruments for depression and anxiety following stroke: experience in the Perth community stroke study. Acta Psychiatrica Scandinavica. 1995;91(4):252–7. 10.1111/j.1600-0447.1995.tb09778.x 7625207 10.1111/j.1600-0447.1995.tb09778.x

[39] Sagen U, Vik TG, Moum T, Mørland T, et al. Screening for anxiety and depression after stroke: comparison of the hospital anxiety and depression scale and the Montgomery and Åsberg depression rating scale. J Psychosom Res. 2009;67(4):325–32. 10.1016/j.jpsychores.2009.03.007 19773025 10.1016/j.jpsychores.2009.03.007

[40] Mitchell AJ, Sheth B, Gill J, et al. Prevalence and predictors of poststroke mood disorders: A meta-analysis and meta-regression of depression, anxiety and adjustment disorder. Gen Hosp Psychiatry. 2017;47:48–60. 10.1016/j.genhosppsych.2017.04.001 28807138 10.1016/j.genhosppsych.2017.04.001

[41] Chan LG, Ng MH, Chan OH, Eu JL, Bajpai R. Clinical impact of a poststroke depression screening program. PM&R. 2025 Apr 21. 10.1002/pmrj.13372 40257226

